# Multimorbidity and its Associated Factors in Korean Shift Workers: Population-Based Cross-Sectional Study

**DOI:** 10.2196/55014

**Published:** 2024-06-10

**Authors:** Hye Chong Hong, Young Man Kim

**Affiliations:** 1 Department of Nursing Chung-Ang University Seoul Republic of Korea; 2 College of Nursing Jeonbuk National University Jeonju Republic of Korea; 3 Research Institute of Nursing Science Jeonbuk National University Jeonju Republic of Korea; 4 Biomedical Research Institute Jeonbuk National University Hospital Jeonju Republic of Korea

**Keywords:** chronic disease, multimorbidity, shift work schedule, shift workers, population-based study, Korea, network analysis, logistic regression, cross-sectional study, public health

## Abstract

**Background:**

Multimorbidity is a crucial factor that influences premature death rates, poor health, depression, quality of life, and use of health care. Approximately one-fifth of the global workforce is involved in shift work, which is associated with increased risk for several chronic diseases and multimorbidity. About 12% to 14% of wage workers in Korea are shift workers. However, the prevalence of multimorbidity and its associated factors in Korean shift workers are rarely reported.

**Objective:**

This study aimed to assess multimorbidity prevalence, examine the factors associated with multimorbidity, and identify multimorbidity patterns among shift workers in Korea.

**Methods:**

This study is a population-based cross-sectional study using Korea National Health and Nutrition Examination Survey data from 2016 to 2020. The study included 1704 (weighted n=2,697,228) Korean shift workers aged 19 years and older. Multimorbidity was defined as participants having 2 or more chronic diseases. Demographic and job-related variables, including regular work status, average working hours per week, and shift work type, as well as health behaviors, including BMI, smoking status, alcohol use, physical activity, and sleep duration, were included in the analysis. A survey-corrected logistic regression analysis was performed to identify factors influencing multimorbidity among the workers, and multimorbidity patterns were identified with a network analysis.

**Results:**

The overall prevalence of multimorbidity was 13.7% (302/1704). Logistic regression indicated that age, income, regular work, and obesity were significant factors influencing multimorbidity. Network analysis results revealed that chronic diseases clustered into three groups: (1) cardiometabolic multimorbidity (hypertension, dyslipidemia, diabetes, coronary heart disease, and stroke), (2) musculoskeletal multimorbidity (arthritis and osteoporosis), and (3) unclassified diseases (depression, chronic liver disease, thyroid disease, asthma, cancer, and chronic kidney disease).

**Conclusions:**

The findings revealed that several socioeconomic and behavioral factors were associated with multimorbidity among shift workers, indicating the need for policy development related to work schedule modification. Further organization-level screening and intervention programs are needed to prevent and manage multimorbidity among shift workers. We also recommend longitudinal studies to confirm the effects of job-related factors and health behaviors on multimorbidity among shift workers in the future.

## Introduction

Shift work includes any work schedule that is outside of the conventional 7 AM to 6 PM working hours. Shift work is prevalent and inevitable in some workforces, including health care, law enforcement, and manufacturing [1]. Globally, a total of approximately one-fifth of the workforce is involved in shift work [2]. While shift work allows workplace flexibility and may provide economic benefits, it may also be associated with adverse chronic health outcomes [3].

Shift work is known to disrupt circadian rhythms and affect sleep patterns, hormone secretion, and other biological processes [4]. Furthermore, these disruptions have been associated with chronic diseases among shift workers, such as metabolic disorders, diabetes, cardiovascular disease, stroke, cancer, and depression [5,6]. However, most epidemiological studies and systematic reviews examining the relationships between shift work and chronic diseases only examined the effect of shift work on a single chronic disease, despite the potential for chronic disease comorbidities. Few studies have examined the coexistence of chronic disease and shift work. For example, Yang et al [7] examined shift work and the risk of cardiometabolic multimorbidity among patients with hypertension and found that shift work was associated with cardiometabolic multimorbidity. In the Korean population, shift work was found to be associated with mental health problems, such as depression and suicide ideation in electronics workers [8], chronic kidney disease in female manual laborers [9], and metabolic syndrome in female workers [10].

Multimorbidity refers to the existence of 2 or more chronic diseases in an individual [11,12]. Unlike comorbidity, which refers to the combined effects of chronic diseases related to a primary chronic disease, multimorbidity examines all chronic diseases simultaneously, which means that no single condition is more important than any other [13]. Multimorbidity is person centered and does not assign priority to a single condition [14]. Worldwide, approximately 37% of the general population has multimorbidity [15], and it is associated with premature death [16], poor health [17,18], depression [19], poor quality of life [20], and increased use of health care [21]. Additionally, age, gender, educational level, smoking, and obesity were associated with multimorbidity in previous research among adults aged 50 years and older [22]. Multimorbidity has become increasingly important as changing health behaviors, such as physical activity and obesity, are a core focus in multimorbidity prevention. Screening and behavioral changes, as well as developing intervention programs, may be important for prevention among people with multimorbidity.

About 12% to 14% of wage workers in Korea are shift workers, and weekly working hours vary from 50 hours to 58 hours depending upon the shift types, which is significantly higher than for day workers [23]. Shift work and long working hours are well-known risk factors for several chronic diseases. However, the prevalence of multimorbidity and its associated factors in Korean shift workers are rarely reported. Identifying and understanding the prevalence of multimorbidity may yield important information for focused care of shift workers and help develop policies related to shift work schedules in Korea, as well as interventions needed to prevent and manage shift workers’ multimorbidity. We additionally performed a network analysis to determine multimorbidity patterns, as certain chronic diseases are likely to co-occur because their pathophysiological pathways are similar [24]. Understanding the patterns of these co-occurring chronic diseases may be beneficial as it may provide vital information for clinicians and policy makers to develop and implement intervention programs for specific groups with similar multimorbidity. Therefore, the purposes of this study are to (1) assess the prevalence of multimorbidity, (2) examine the factors associated with multimorbidity among shift workers in Korea, and (3) identify patterns of multimorbidity.

## Methods

This study follows the Strengthening the Reporting of Observational Studies in Epidemiology (STROBE) guidelines [25].

### Design

This is a population-based, cross-sectional study using Korea National Health and Nutrition Examination Survey (KNHANES) data from 2016 to 2020.

### Data Source

KNHANES uses a nationally representative sample based on a complex, stratified, multistage cluster sampling method that includes geographical area, gender, and age to provide a representation of the Korean population that is freely available to the public. KNHANES uses a series of cross-sectional national surveys that have been conducted by the Korean Centers for Disease Control and Prevention. These consist of a health survey, a health examination, and a nutrition survey [26].

### Study Sample

The participants of this study were shift workers aged 19 years and older. Shift workers were defined as those who work at night or are nonday workers. This included both evening work, night work, day/night regular shift work, and irregular shift work. The exclusion criteria were as follows: (1) those who were younger than 19 years, (2) those who were nonworkers, and (3) those with missing data on any of the chronic diseases. The detailed sample selection flow is shown in Multimedia Appendix 1, Figure S1.

### Ethical Considerations

This study was exempted from ethical review and approval by the institutional review board of Chung-Ang University, HCH’s institution (1041078-20230518-HR-139), as this study was a secondary analysis of preexisting data. The primary data (from KNHANES) were anonymous, and informed consent was obtained prior to the data collection.

### Measures

#### Multimorbidity

We defined multimorbidity as participants having 2 or more chronic conditions simultaneously. Based on previous studies [27-29], 13 common worldwide chronic diseases were included. These included hypertension, dyslipidemia, diabetes, arthritis, cancer, asthma, depression, osteoporosis, thyroid disease, coronary heart disease (CHD), chronic liver disease (CLD), stroke, and chronic kidney disease (CKD). The presence of disease was determined by self-reporting by the participants as to whether they had ever been diagnosed with each disease by a physician. The multimorbidity group was operationally defined as shift workers with 2 or more of the 13 diseases. The nonmultimorbidity group was defined as healthy participants or participants with only 1 chronic condition among the 13 diseases.

#### Demographic and Job-Related Variables

Sex, age, household income, education, and marital status were considered as demographic characteristics. Household income was divided into quartiles, which were calculated annually to evenly distribute the population into 4 groups by sex and age using the monthly average equivalized income. Education was divided into 4 groups: elementary school or less, middle school, high school, and university and above. Marital status was categorized as married or single. Job-related variables included regularity of work and average working hours per week. The type of shift work was divided into evening work, night work, regular shift work, and irregular shift work. Regular work was defined as that conducted by permanent workers or full-time employees, while nonregular work included work conducted by contract workers, contractors, and part-time workers.

#### Health Behaviors

This study used multiple health behavior variables, including BMI, smoking status, alcohol use, physical activity, and poor sleep duration. BMI was categorized as underweight (<18.5 kg/m^2^), normal (≥18.5 to <23 kg/m^2^), overweight (≥23 to <25 kg/m^2^), and obese (≥25 kg/m^2^) based on the World Health Organization cutoffs for Asia-Pacific countries [30]. Smoking status was categorized as nonsmoker, ex-smoker, and current smoker. Alcohol use was categorized into low-risk drinking, at-risk drinking, alcohol abuse, and alcohol dependence using the Korean version of the Alcohol Use Disorders Identification Test–Concise (AUDIT-C) [31]. Physical activity was measured by aerobic physical activity rate. If participants exercised for at least 2 hours and 30 minutes of moderate-intensity physical activity per week, or at least 1 hour and 15 minutes of high-intensity physical activity, or a mixture of moderate and high-intensity physical activities (1 min of high-intensity activity equals 2 min of moderate-intensity activity), then it was counted as “yes” for aerobic physical activity. Poor sleep duration was defined as either less than 7 hours or more than 9 hours per day; therefore, 7 to 9 hours of sleep per day was considered a good sleep duration [32,33].

### Data Analysis

Data were analyzed using Stata (version 16.1; StataCorp LLC) and JASP (version 0.17.1; Eric-Jan Wagenmakers). Additionally, the KNHANES analytic guidelines were followed to adjust the data for complex sampling designs to estimate the population-level statistics for Korea. We conducted a complete case analysis without any imputation following the analytic guidelines [26]. All statistical tests were based on point estimation using a 2-sided *P* value (<.05) and interval estimation using 95% CIs. Specific analyses was conducted as follows: first, descriptive statistics were used, including weighted N, N, weighted percentage, weighted mean, and SE to present the population characteristics and their multimorbidity characteristics. Second, univariate analyses using the survey-corrected Rao-Scott *χ*^2^ test and adjusted Wald test were conducted to reveal the differing characteristics between the multimorbidity and the nonmultimorbidity shift worker groups. Third, survey-corrected logistic regression analyses were used to identify factors influencing multimorbidity among shift workers. In the final model, variables that were statistically significant in the univariate analysis were included. We additionally conducted a subgroup analysis based on age, using 50 years as the threshold. Lastly, network analysis was used for exploring the patterns of multimorbidity clusters among shift workers. The network model was graphically represented by nodes (circles representing each morbidity) and edges (lines connecting the nodes). The structural importance of multimorbidity patterns was analyzed using node centrality measures, including closeness, betweenness, strength, and expected influence. Clustering and naming of subgroups within multimorbidity groups were determined through discussion among the researchers based on prior studies [7,34].

## Results

### Participant Characteristics

The final selected shift worker sample size was 1704 and the weighted population size was 2,697,228. The weighted percentage of male workers (56.7%) was slightly more than female workers, and the mean age of the participants was 41.93 (SE 0.42; SD 13.74) years, with an age range of 19 to 80 years (Table 1). The majority of the participants had household incomes in the third (n=538, 32.9%) and fourth (n=561, 33.4%) quartiles. As for educational attainment levels, 87.2% (n=1419) of participants had a high school education or higher. Additionally, there were more married (n=1209, 64.6%) than single participants. Participants were divided by shift work type: 920 (53.4%) in evening work, 191 (12.2%) in night work, 454 (27.3%) in regular shift work, and 139 (7.1%) in irregular shift work. The proportion of nonregular workers was high at 63.7% (n=821), and the average number of working hours per week was 38.01 (SD 17.67).

**Table 1:** General characteristics of shift workers (N=1704; weighted N=2,697,228).

Characteristics			Values
**Demographics**			
	**Sex, n (weighted %)**		
		Male	849 (56.7)
		Female	855 (43.3)
	Age (years), mean (SE; SD)		41.93 (0.42; 13.74)
	**House income (quartile), n (weighted %)**		
		Low (first)	170 (9.3)
		Lower middle (second)	432 (25.4)
		Higher middle (third)	538 (32.9)
		High (fourth)	561 (33.4)
	**Education, n (weighted %)**		
		≤Elementary school	153 (5.7)
		Middle school	132 (6.1)
		High school	743 (46.4)
		≥University	676 (41.8)
	**Marital status, n (weighted %)**		
		Married	1209 (64.6)
		Single	495 (35.4)
**Occupational status**			
	**Shift work type, n (weighted %)**		
		Evening work	920 (53.4)
		Night work	191 (12.2)
		Regular shift work	454 (27.3)
		Irregular shift work	139 (7.1)
	**Regular work, n (weighted %)**		
		Yes	417 (36.3)
		No	821 (63.7)
	Working hours per week, mean (SE; SD)		38.01 (0.59; 17.67)
**Health**			
	**BMI**		
		Overall (kg/m^2^), mean (SE; SD)	24.20 (0.11; 3.47)
		Normal, n (weighted %)	618 (35.7)
		Underweight, n (weighted %)	70 (4)
		Overweight, n (weighted %)	410 (23.6)
		Obese, n (weighted %)	600 (36.7)
	**Smoking, n (weighted %)**		
		Nonsmoker	954 (51.9)
		Ex-smoker	331 (19.9)
		Current smoker	419 (28.2)
	**Alcohol use, n (weighted %)**		
		Low-risk drinking	969 (67.2)
		At-risk drinking	133 (10.5)
		Alcohol abuse	192 (15.2)
		Alcohol dependence	84 (7.1)
	**Physical activity**		
		Yes, n (weighted %)	808 (50.05)
		No, n (weighted %)	890 (49.95)
	Sleep hours, mean (SE; SD)		7.34 (0.04; 1.33)
	**Poor sleep, n (weighted %)**		
		Yes (<7 h or >9 h)	940 (55.9)
		No (7 to 9 h)	758 (44.1)

The mean BMI was 24.20 (SE 0.11; SD 3.47) kg/m^2^, and overweight and obese participants (those with a BMI of 23.0 kg/m^2^ or higher) accounted for 60.3% of the total. Nonsmokers were the most common at 51.9% (n=954), and low-risk drinking was engaged in by the majority of the workers at 67.2% (n=969). The proportion of workers engaged in aerobic physical activity was 50.05% (n=808), which was similar to the proportion of participants not engaged in aerobic physical activity. The average sleep time was 7.34 (SD 1.33) hours, and workers who showed a poor sleep pattern accounted for the majority at 55.9% (n=940).

### Multimorbidity Characteristics of Shift Workers

Table 2 shows the multimorbidity characteristics of the shift workers. Hypertension (n=287, 14.2%) was the most common of the 13 chronic conditions, followed by dyslipidemia (n=235, 11%), diabetes (n=107, 5.3%), and arthritis (n=126, 5.1%). The multimorbidity prevalence in shift workers was found to be 13.7% (n=302) and the number of chronic conditions ranged from 0 to 6.

**Table 2:** Multimorbidity characteristics of shift workers (N=1704; weighted N=2,697,228).

Variables		Workers, n (weighted %)
**Chronic conditions^a^**		
	Hypertension	287 (14.2)
	Dyslipidemia	235 (11)
	Diabetes	107 (5.3)
	Arthritis	126 (5.1)
	Cancer	90 (3.9)
	Asthma	50 (3.3)
	Depression	60 (3.1)
	Osteoporosis	68 (2.7)
	Thyroid disease	52 (2.7)
	Coronary heart disease	33 (1.3)
	Chronic liver disease	14 (0.9)
	Stroke	12 (0.5)
	Chronic kidney disease	10 (0.5)
**Number of chronic conditions^b^**		
	Zero	1046 (67.1)
	One	356 (19.2)
	Two	175 (8.3)
	Three	84 (3.8)
	Four	32 (1.2)
	Five	8 (0.3)
	Six	3 (0.1)

^a^The order of chronic conditions is from the highest weighted percentage to the lowest.

^b^The average number of chronic conditions was 0.54 (SE 0.02; SD 0.87; range 0-6 for the total of 13 chronic conditions).

### Differences in Characteristics by Presence of Multimorbidity

The differences between the multimorbidity group and the nonmultimorbidity group are presented in Table 3. The average age of the multimorbidity group was 57.21 (SE 0.69; SD 10.47) years, which was statistically significantly higher than that of the nonmultimorbidity group (mean 39.51, SE 0.42, SD 12.69 years). The multimorbidity group had lower household income and educational level, a lower number of unmarried people, higher irregular work level, higher BMI, fewer current smokers, less physical activity, and a higher proportion of participants with poor sleep compared to the nonmultimorbidity group, with all differences being statistically significant.

**Table 3:** Differences in characteristics of the multimorbidity and nonmultimorbidity groups of shift workers (N=1704; weighted N=2,697,228).

Characteristics		Nonmultimorbidity (n=1402, 86.3%)		Multimorbidity (n=302, 13.7%)		*F* test (*df*)		*P* value	
**Sex, n (weighted %)**							2.71 (862)		.10
	Male	710 (49.7)	139 (7.1)						
	Female	139 (36.6)	163 (6.6)						
Age, mean (SE)		39.51 (0.42)		57.21 (0.69)		497.79^a^ (862)		<.001	
**Age group (years), n (weighted %)**							240.28 (862)		<.001
	<50	956 (64.4)	45 (2.7)						
	≥50	446 (21.9)	257 (11)						
**House income (quartile), n (weighted %)**							10.03 (862)		<.001
	Low (first)	113 (6.9)	58 (2.4)						
	Lower middle (second)	330 (20.3)	102 (4.1)						
	Higher middle (third)	458 (28.9)	80 (4)						
	High (fourth)	499 (30.2)	62 (3.2)						
**Education, n (weighted %)**							55.52 (862)		<.001
	≤Elementary school	67 (2.7)	86 (3.0)						
	Middle school	82 (4.1)	50 (2.0)						
	High school	632 (40.8)	111 (5.6)						
	≥University	621 (38.7)	55 (3.1)						
**Marital status, n (weighted %)**							92.36 (862)		<.001
	Married	922 (51.7)	287 (12.9)						
	Single	480 (34.6)	15 (0.8)						
**Shift work type, n (weighted %)**							1.59 (862)		.19
	Evening work	769 (46.4)	151 (7.0)						
	Night work	162 (10.9)	29 (1.3)						
	Regular shift work	369 (23.1)	85 (4.2)						
	Irregular shift work	102 (5.8)	37 (1.3)						
**Regular work, n (weighted %)**							8.10 (862)		.005
	Yes	369 (33.0)	48 (3.3)						
	No	652 (53.7)	169 (10.0)						
Working hours per week, mean (SE)		37.90 (0.62)		38.72 (1.42)		0.30^a^ (862)		.58	
BMI, mean (SE)		23.98 (0.12)		25.57 (0.24)		34.45^a^ (862)		<.001	
**BMI category, n (weighted %)**							11.87 (862)		<.001
	Normal	536 (32.5)	82 (3.3)						
	Underweight	67 (3.9)	3 (0.1)						
	Overweight	330 (19.9)	80 (3.7)						
	Obese	463 (30.0)	137 (6.7)						
**Smoking, n (weighted %)**							5.90 (862)		.003
	Nonsmoker	780 (44.7)	174 (7.2)						
	Ex-smoker	257 (16.1)	74 (3.8)						
	Current smoker	365 (25.5)	54 (2.7)						
**Alcohol use, n (weighted %)**							1.19 (862)		.31
	Low risk drinking	830 (59.5)	139 (7.7)						
	At-risk drinking	116 (9.5)	17 (1)						
	Alcohol abuse	169 (13.7)	23 (1.5)						
	Alcohol dependence	67 (5.9)	17 (1.2)						
**Physical activity**							5.50 (862)		.02
	Yes	688 (44.2)	120 (5.8)						
	No	708 (42.0)	182 (7.9)						
**Poor sleep**							10.21 (862)		.002
	Yes (<7 h or >9 h)	595 (36.6)	163 (7.5)						
	No (7-9 h)	801 (49.7)	139 (6.2)						

^a^Adjusted Wald test.

### Influencing Factors for Multimorbidity in Shift Workers

Table 4 presents the factors affecting multimorbidity in shift workers. Examining the relationship between each influencing factor and the 2 variables (crude odds ratio [OR]) of multimorbidity showed that age, household income, education, marital status, regularity of work, BMI, smoking, physical activity, and poor sleep quality were all factors influencing multimorbidity. In the final adjusted logistic model, factors affecting multimorbidity were age (OR 1.106, 95% CI 1.069-1.144; *P*<.001) third-quartile (OR 0.302, 95% CI 0.119-0.768; *P*=.01) vs first-quartile household income, fourth-quartile (OR 0.366, 95% CI 0.142-0.942; *P*=.04) vs first-quartile household income, nonregular work (OR 1.804, 95% CI 1.008-3.228; *P*=.047), and obese BMI (OR 2.152, 95% CI 1.155-4.010; *P*=.02) vs normal BMI. That is, when the age increased by 1 year, the risk of multimorbidity increased by 1.11 times, and the risk of multimorbidity in the third-quartile and fourth-quartile household income groups decreased by 69.8% and 63.4%, respectively, compared to the first-quartile household income group. The risk of multimorbidity in the nonregular work group increased by 1.8 times compared with the regular work group, and the risk of multimorbidity in the obese group increased by 2.15 times compared to the normal BMI group. Subgroup analysis showed that age, regular work, and obese BMI were associated with multimorbidity in the subgroup younger than 50 years, while household income, education, marital status, and obese BMI were associated in the subgroup aged 50 years and older (Multimedia Appendix 2, Table S1).

**Table 4:** Factors affecting multimorbidity in shift workers (N=1704; weighted N=2,697,228).

Factors		Crude OR^a^ (95% CI)	*P* value	Adjusted OR (95% CI)	*P* value
Age		1.106 (1.091-1.120)	<.001	1.106 (1.069-1.144)	<.001
**House income (quartiles)**					
	Low (first)	Reference	—^b^	—	—
	Lower middle (second)	0.597 (0.378-0.943)	.03	0.467 (0.193-1.129)	.09
	Higher middle (third)	0.406 (0.261-0.630)	<.001	0.302 (0.119-0.768)	.01
	High (fourth)	0.311 (0.195-0.496)	<.001	0.366 (0.142-0.942)	.04
**Education**					
	≤Elementary school	Reference	—	—	—
	Middle school	0.439 (0.257-0.750)	.003	0.503 (0.202-1.255)	.14
	High school	0.126 (0.082-0.196)	<.001	0.611 (0.293-1.276)	.19
	≥University	0.074 (0.046-0.119)	<.001	0.528 (0.213-1.304)	.17
**Marital status**					
	Married	Reference	—	—	—
	Single	0.089 (0.049-0.164)	<.001	0.749 (0.245-2.292)	.61
**Regular work**					
	Yes	Reference	—	—	—
	No	1.847 (1.199-2.847)	.006	1.804 (1.008-3.228)	.047
**BMI**					
	Normal	Reference	—	—	—
	Underweight	0.251 (0.074-0.859)	.03	—	—
	Overweight	1.815 (1.261-2.612)	.001	0.918 (0.466-1.809)	.81
	Obese	2.216 (1.581-3.106)	<.001	2.152 (1.155-4.010)	.02
**Smoking**					
	Nonsmoker	Reference	—	—	—
	Ex-smoker	1.472 (1.025-2.115)	.04	0.950 (0.474-1.903)	.89
	Current smoker	0.668 (0.446-0.999)	.05	0.987 (0.490-1.990)	.97
**Physical activity**					
	No	Reference	—	—	—
	Yes	0.701 (0.516-0.951)	.02	1.435 (0.819-2.514)	.21
**Poor sleep**					
	No (7-9 h)	Reference	—	—	—
	Yes (<7 h or >9 h)	1.650 (1.213-2.245)	.001	1.467 (0.880-2.445)	.14

^a^OR: odds ratio.

^b^Not applicable.

### Network Analysis

**Figure 1:**
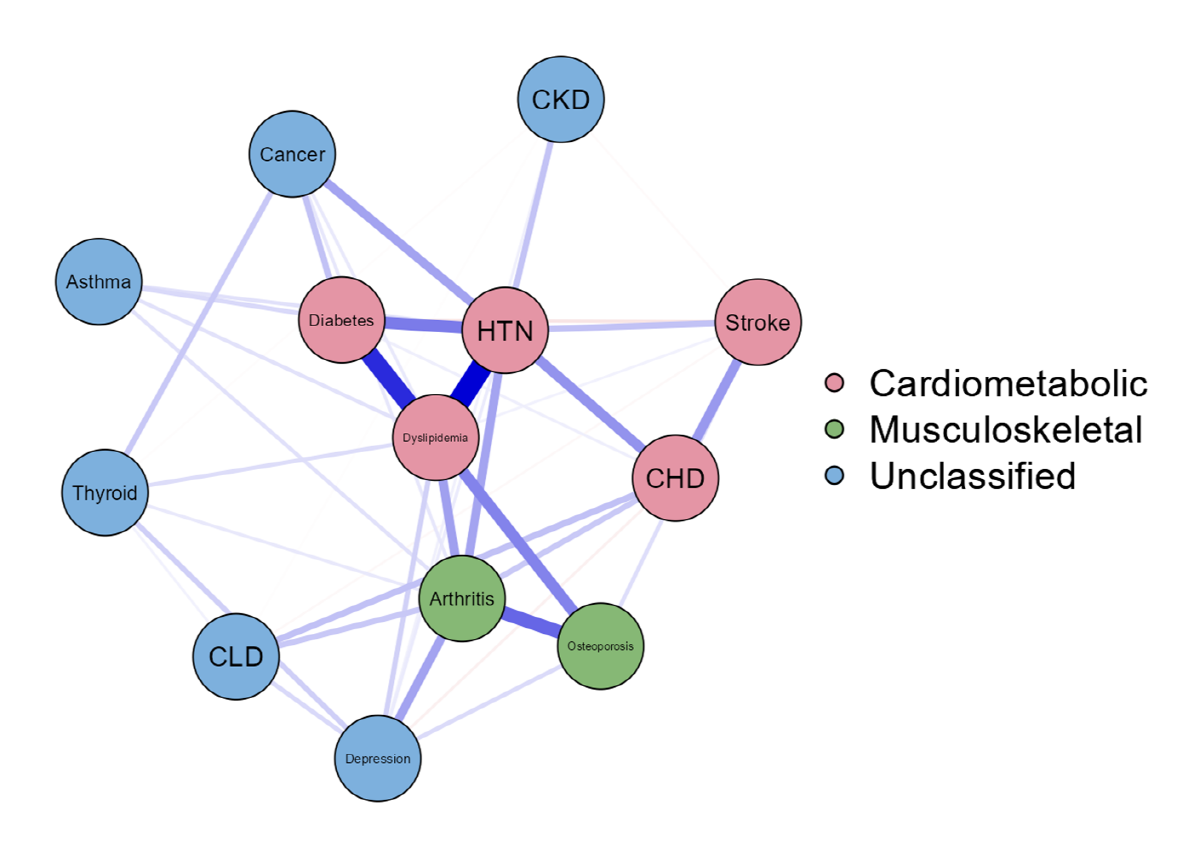
Multimorbidity network in shift workers. CKD: chronic kidney disease; HNT: hypertension; CHD: coronary heart disease; CLD: chronic liver disease.

As presented in Figure 1, the network analysis revealed that chronic diseases were clustered into three groups: (1) cardiometabolic multimorbidity (hypertension, dyslipidemia, diabetes, CHD, and stroke), (2) musculoskeletal multimorbidity (arthritis and osteoporosis), and (3) unclassified diseases (depression, CLD, thyroid disease, asthma, cancer, and CKD). A centrality plot of multimorbidity patterns in shift workers shows hypertension, dyslipidemia, arthritis, diabetes, and osteoporosis by strength and expected influence in order (Multimedia Appendix 3, Figure S2).

## Discussion

### Overview

This study aimed to identify patterns of multimorbidity and examine the factors associated with multimorbidity among shift workers in Korea. Among the 1704 shift workers included in the study, approximately 14% had multimorbidity. A recent meta-analysis of multimorbidity in the worldwide adult population found that there was a prevalence of approximately 37% [15]. The difference in prevalence could be due to several factors. First, the mean age of our sample was relatively low at 41.9 (SD 13.7) years, compared to 56.9 years in the meta-analysis. It has been recognized that older age is associated with an increased number of chronic diseases [35]. Furthermore, women are more prone to developing chronic diseases than men [36,37], and a higher proportion of our sample was male.

It is recognized that shift work is associated with adverse health outcomes. Previous research reported that shift work increases the risk of cardiovascular incidence and mortality, cancer, and stroke [1,7,38-40]. Approximately 14% of the Korean population is involved in shift work, and although some shift work is inevitable and may provide economic benefits, policies are needed to modify and manage shift schedules. Socioeconomic and behavioral factors are also known to increase the risk for multimorbidity. Consistent with previous research, we found that shift workers in the low-income and obese groups had increased risks of developing multimorbidity. The relationship between low income and the development of chronic diseases and multimorbidity may be explained by less physical activity and lower fruit and vegetable consumption in the low-income group [41,42]. Furthermore, obesity is a major risk factor associated with developing several chronic diseases, including diabetes, heart disease, asthma, arthritis, and depression [43]. We found that a high proportion of Korean workers are involved in nonregular work (63.7%), and having regular work was a significant factor influencing multimorbidity. Many nonregular workers are paid low wages (less than US $10 per hour), are employed on 2-year fixed-term contracts, and may be required to extend their employment or ask to terminate the employment every 2 years. Those who work less than a certain number of hours per week are not covered by social insurance and are not entitled to weekly holidays or paid annual leave, which could be important factors contributing to poor health outcomes among nonregular workers in Korea [44]. Labor laws may require reform so that nonregular workers are guaranteed stability in their jobs, social insurance coverage, and proper wages to enable a minimum standard of living. However, longitudinal and cohort studies are needed to assess and analyze the relationships between shift work, socioeconomic and behavioral factors, and multimorbidity. Further, the subgroup analysis revealed that education and marital status were significant factors influencing multimorbidity in shift workers aged 50 years and older. Consistent with previous research, the risk of multimorbidity increased when workers were less educated and when they were single [45,46]. A deeper understanding of these relationships may require further analysis to identify the subgroups at greatest risk for multimorbidity and develop targeted interventions, as there could be interactions between socioeconomic and demographic factors.

The most common chronic disease in this study was hypertension, followed by dyslipidemia, diabetes, and arthritis. This is similar to the findings of a previous meta-analysis of multimorbidity in the United States, United Kingdom, and Spain [20]. Additionally, we performed a network analysis and found 2 distinct groups: hypertension, dyslipidemia, diabetes, CHD, and stroke clustered as one group; arthritis and osteoporosis clustered as another group. Previous research found that the coexistence of hypertension and at least 1 other comorbidity was most common among patients with multimorbidity [47]. Similar to our findings, Yang et al [7] found that shift workers with hypertension in the United Kingdom were more prone to cardiometabolic multimorbidity, including diabetes, coronary artery disease, and stroke. However, the only known multimorbidity cluster is cardiovascular multimorbidity, and the evidence on multimorbidity in other chronic diseases and their clusters in shift workers remains limited. In the general population, cardiovascular diseases and metabolic diseases tend to cluster together, and osteoarthritis tended to cluster together in an Australian sample, a finding that is similar to ours [48]. Some chronic diseases are more likely to cluster together, as seen in our study and the studies in the United Kingdom and Australia. Some chronic diseases are more likely to cluster together as they may share similar pathophysiological pathways [24]. Also, shift work is known to disrupt circadian rhythms and sleep and is associated with overweight and with blood glucose levels, which are important factors for developing cardiometabolic diseases [49,50]. Although it is controversial, some have suggested that patients with arthritis may have greater bone loss [51]. Considering the relationship between arthritis and osteoporosis and that the incidence of both conditions increases with age [52,53], and that the mean age of our participants with multimorbidity was 57 (SD 10.47) years, the co-occurrence of these chronic disease may be understandable. Identifying these disease clusters can be important, as we can target and tailor interventions for specific groups. However, caution is needed when interpreting and comparing clusters of multimorbidity or patterns across studies, as researchers use different statistical methods to analysis clusters or patterns and may include a different list of chronic diseases in their analyses. Further research is needed to better understand and assess multimorbidity clusters and their trajectories and patterns over time among shift workers using unified and valid analytic methods with the most prevalent chronic diseases in shift workers.

Most chronic care models and guidelines focus on the treatment and management of individual chronic diseases [54]. Multimorbidity requires more complex care that prioritizes what is most important for each patient. Several types of interventions for multimorbidity have been implemented in the past, but the evidence to deliver specific interventions remains limited. Furthermore, interventions specific for shift workers with multimorbidity are relatively unknown, despite shift workers being at greater risk for developing multimorbidity than nonshift workers. Nevertheless, occupational or organization-based interventions may benefit shift workers. These potentially include policy development regarding shift schedules, early screening diagnostic interventions, and diet and physical activity programs. However, additional research remains necessary to confirm the multimorbidity clusters in shift workers and to confirm the differences between socioeconomic and behavioral factors to enable the development of specific intervention programs for each cluster group.

To our knowledge, this study is the first to assess multimorbidity and its associated factors in Korean shift workers. We analyzed a 2016-2020 series of national population-based study data (KNHANES) using network analysis to reveal multimorbidity patterns. However, this study has some limitations. First, KNHANES is a cross-sectional study; thus, we cannot assume temporal relationships between variables. We also could not assess the long-term patterns of multimorbidity development and the effect of socioeconomic behavioral factors on multimorbidity. Therefore, we recommend longitudinal studies on multimorbidity among shift workers in the future. Second, the data were based on self-reports from participants. Thus, recall bias cannot be excluded. Some objective data, for example, medical records to identify multimorbidity, may be needed in a future study. Third, the specific patterns of shift work were not specified in the current data set. For example, we do not know if the shift workers had rotating shifts, only night shifts, or how many night or evening shifts were included in their work schedules. Further studies should specify the shift work schedules; a subgroup analysis may be beneficial. Fourth, we also recommend subgroup analysis of different work sectors, since working conditions and shift schedules may differ. For example, the health care and manufacturing industries may use more complex shift schedules and require more specific interventions based on subgroup analysis. Fifth, network analysis provides only a graphical presentation of multimorbidity patterns and does not allow for statistical analyses, such as regression, to identify factors associated with each multimorbidity pattern. Future studies may consider using other statistical analyses, such as latent class analysis, if researchers are interested in exploring factors associated with multimorbidity patterns in shift workers. Finally, the study results may have excluded some confounding variables, such as family support and use of health care, that may have influenced multimorbidity.

### Conclusion

Multimorbidity is a crucial factor influencing premature death, poor health and quality of life, and use of health care. Our findings indicate that approximately 14% of Korean shift workers have multimorbidity and that several socioeconomic and behavioral factors are associated with multimorbidity. This suggests that policy development regarding work schedule modification is necessary. Furthermore, screening and tailored intervention programs at the organizational level may benefit efforts to prevent and monitor multimorbidity among shift workers. However, we also suggest a future longitudinal study to assess and confirm multimorbidity patterns among shift workers.

## Appendix

Multimedia Appendix 1 Flow diagram of study sample selection.

Multimedia Appendix 2 Age-subgroup analysis of factors affecting multimorbidity in shift workers.

Multimedia Appendix 3 Centrality plot of multimorbidity patterns in shift workers presenting hypertension, dyslipidemia, arthritis, diabetes, and osteoporosis according to strength and expected influence in order.

## Abbreviations

AUDIT-C: Alcohol Use Disorders Identification Test-Concise
CHD: coronary heart disease
CLD: chronic liver disease
CKD: chronic kidney disease
KNHANES: Korea National Health and Nutrition Examination Survey
OR: odds ratio
STROBE: Strengthening the Reporting of Observational Studies in Epidemiology
WHO: World Health Organization

## Data Availability

The data supporting the findings of this study are derived from the Korea National Health and Nutrition Examination Survey (KNHANES), which is publicly accessible at no cost. Interested researchers can access the data by visiting the KNHANES website [55]. Please note that while the data are freely available, users are encouraged to comply with the terms and conditions specified on the KNHANES portal.

## References

[1] Rivera AS, Akanbi M, O'Dwyer LC, McHugh M (2020). Shift work and long work hours and their association with chronic health conditions: A systematic review of systematic reviews with meta-analyses. PLoS One.

[2] Messenger J (2018). Working time and the future of work. International Labour Organization.

[3] Crowther ME, Ferguson SA, Vincent GE, Reynolds AC (2021). Non-pharmacological interventions to improve chronic disease risk factors and sleep in shift workers: a systematic review and meta-analysis. Clocks Sleep.

[4] Touitou Y, Reinberg A, Touitou D (2017). Association between light at night, melatonin secretion, sleep deprivation, and the internal clock: Health impacts and mechanisms of circadian disruption. Life Sci.

[5] Haus EL, Smolensky MH (2013). Shift work and cancer risk: potential mechanistic roles of circadian disruption, light at night, and sleep deprivation. Sleep Med Rev.

[6] Moreno CRC, Marqueze EC, Sargent C, Wright Jr KP, Ferguson SA, Tucker P (2019). Working Time Society consensus statements: evidence-based effects of shift work on physical and mental health. Ind Health.

[7] Yang L, Luo Y, He L, Yin J, Li T, Liu S, Li D, Cheng X, Bai Y (2022). Shift work and the risk of cardiometabolic multimorbidity among patients with hypertension: a prospective cohort study of UK Biobank. J Am Heart Assoc.

[8] Kang M, Kwon H, Choi K, Kang C, Kim H (2017). The relationship between shift work and mental health among electronics workers in South Korea: A cross-sectional study. PLoS One.

[9] Uhm JY, Kim H, Kang GH, Choi YG, Park TH, Kim SY, Chang SS, Choo WO (2018). The association between shift work and chronic kidney disease in manual labor workers using data from the Korea National Health and Nutrition Examination Survey (KNHANES 2011-2014). Ann Occup Environ Med.

[10] Yu KH, Yi YH, Kim YJ, Cho BM, Lee SY, Lee JG, Jeong DW, Ji SY (2017). Shift work is associated with metabolic syndrome in young female Korean workers. Korean J Fam Med.

[11] van den Akker M, Buntinx F, Metsemakers JF, Roos S, Knottnerus J (1998). Multimorbidity in general practice: prevalence, incidence, and determinants of co-occurring chronic and recurrent diseases. J Clin Epidemiol.

[12] Mercer S, Furler J, Moffat K, Fischbacher-Smith D, Sanci L (2016). Multimorbidity: Technical Series on Safer Primary Care.

[13] Tugwell P, Knottnerus JA (2019). Multimorbidity and comorbidity are now separate MESH headings. J Clin Epidemiol.

[14] Skou ST, Mair FS, Fortin M, Guthrie B, Nunes BP, Miranda JJ, Boyd CM, Pati S, Mtenga S, Smith SM (2022). Multimorbidity. Nat Rev Dis Primers.

[15] Chowdhury SR, Chandra Das D, Sunna TC, Beyene J, Hossain A (2023). Global and regional prevalence of multimorbidity in the adult population in community settings: a systematic review and meta-analysis. EClinicalMedicine.

[16] Menotti A, Mulder I, Nissinen A, Giampaoli S, Feskens EJ, Kromhout D (2001). Prevalence of morbidity and multimorbidity in elderly male populations and their impact on 10-year all-cause mortality: The FINE study (Finland, Italy, Netherlands, Elderly). J Clin Epidemiol.

[17] Sheridan PE, Mair CA, Quiñones Ana R (2019). Associations between prevalent multimorbidity combinations and prospective disability and self-rated health among older adults in Europe. BMC Geriatr.

[18] Tang LH, Thygesen LC, Willadsen TG, Jepsen R, la Cour K, Frølich Anne, Møller Anne, Jørgensen Lars Bo, Skou ST (2020). The association between clusters of chronic conditions and psychological well-being in younger and older people-A cross-sectional, population-based study from the Lolland-Falster Health Study, Denmark. J Comorb.

[19] Read JR, Sharpe L, Modini M, Dear BF (2017). Multimorbidity and depression: A systematic review and meta-analysis. J Affect Disord.

[20] Makovski TT, Schmitz S, Zeegers MP, Stranges S, van den Akker M (2019). Multimorbidity and quality of life: systematic literature review and meta-analysis. Ageing Res Rev.

[21] Pati S, Swain S, Hussain MA, van den Akker M, Metsemakers J, Knottnerus JA, Salisbury C (2015). Prevalence and outcomes of multimorbidity in South Asia: a systematic review. BMJ Open.

[22] Souza DLB, Oliveras-Fabregas A, Minobes-Molina E, de Camargo Cancela M, Galbany-Estragués Paola, Jerez-Roig J (2021). Trends of multimorbidity in 15 European countries: a population-based study in community-dwelling adults aged 50 and over. BMC Public Health.

[23] Jang T (2023). Working hours and the regulations for night shift workers. Ann Occup Environ Med.

[24] Kessler R, Smelser NJ, Baltes PB (2001). Comorbidity. International Encyclopedia of the Social & Behavioral Sciences.

[25] von Elm Erik, Altman DG, Egger M, Pocock SJ, Gøtzsche Peter C, Vandenbroucke JP, STROBE Initiative (2008). The Strengthening the Reporting of Observational Studies in Epidemiology (STROBE) statement: guidelines for reporting observational studies. J Clin Epidemiol.

[26] Kweon S, Kim Y, Jang M, Kim Y, Kim K, Choi S, Chun C, Khang Y, Oh K (2014). Data resource profile: the Korea National Health and Nutrition Examination Survey (KNHANES). Int J Epidemiol.

[27] Ho IS, Azcoaga-Lorenzo A, Akbari A, Black C, Davies J, Hodgins P, Khunti K, Kadam U, Lyons RA, McCowan C, Mercer S, Nirantharakumar K, Guthrie B (2021). Examining variation in the measurement of multimorbidity in research: a systematic review of 566 studies. Lancet Public Health.

[28] Park B, Ock M, Lee HA, Lee S, Han H, Jo M, Park H (2018). Multimorbidity and health-related quality of life in Koreans aged 50 or older using KNHANES 2013-2014. Health Qual Life Outcomes.

[29] Tang F, Gates Kuliszewski M, Carrascal A, Vásquez E (2021). Physical multimorbidity and cancer prevalence in the National Health and Nutrition Examination Survey. Public Health.

[30] The Asia-Pacific perspective: redefining obesity and its treatment. World Health Organization.

[31] Seong J, Lee C, Do H, Oh S, Lym Y, Choi J, Joh H, Kweon K, Cho D (2009). Performance of the AUDIT Alcohol Consumption Questions (AUDIT-C) and AUDIT-K question 3 alone in screening for problem drinking. Korean J Fam Med.

[32] He L, Biddle SJH, Lee JT, Duolikun N, Zhang L, Wang Z, Zhao Y (2021). The prevalence of multimorbidity and its association with physical activity and sleep duration in middle aged and elderly adults: a longitudinal analysis from China. Int J Behav Nutr Phys Act.

[33] Hirshkowitz M, Whiton K, Albert SM, Alessi C, Bruni O, DonCarlos L, Hazen N, Herman J, Adams Hillard PJ, Katz ES, Kheirandish-Gozal L, Neubauer DN, O'Donnell AE, Ohayon M, Peever J, Rawding R, Sachdeva RC, Setters B, Vitiello MV, Ware JC (2015). National Sleep Foundation's updated sleep duration recommendations: final report. Sleep Health.

[34] Batista SR, Sousa ALL, Nunes BP, Silva RR, Jardim PCBV, Brazilian Group of Studies on Multimorbidity (GBEM) (2022). Identifying multimorbidity clusters among Brazilian older adults using network analysis: findings and perspectives. PLoS One.

[35] MacNee W, Rabinovich RA, Choudhury G (2014). Ageing and the border between health and disease. Eur Respir J.

[36] Alswat KA (2017). Gender disparities in osteoporosis. J Clin Med Res.

[37] Ballering AV, Bonvanie IJ, Olde Hartman TC, Monden R, Rosmalen JGM (2020). Gender and sex independently associate with common somatic symptoms and lifetime prevalence of chronic disease. Soc Sci Med.

[38] Ho FK, Celis-Morales C, Gray SR, Demou E, Mackay D, Welsh P, Katikireddi SV, Sattar N, Pell JP (2022). Association and pathways between shift work and cardiovascular disease: a prospective cohort study of 238 661 participants from UK Biobank. Int J Epidemiol.

[39] Vetter C, Devore EE, Wegrzyn LR, Massa J, Speizer FE, Kawachi I, Rosner B, Stampfer MJ, Schernhammer ES (2016). Association between rotating night shift work and risk of coronary heart disease among women. JAMA.

[40] Wang D, Ruan W, Chen Z, Peng Y, Li W (2018). Shift work and risk of cardiovascular disease morbidity and mortality: A dose-response meta-analysis of cohort studies. Eur J Prev Cardiol.

[41] Leung CW, Epel ES, Ritchie LD, Crawford PB, Laraia BA (2014). Food insecurity is inversely associated with diet quality of lower-income adults. J Acad Nutr Diet.

[42] VanKim NA, Laska MN (2012). Socioeconomic disparities in emerging adult weight and weight behaviors. Am J Health Behav.

[43] Keramat SA, Alam K, Rana RH, Chowdhury R, Farjana F, Hashmi R, Gow J, Biddle SJH (2021). Obesity and the risk of developing chronic diseases in middle-aged and older adults: findings from an Australian longitudinal population survey, 2009-2017. PLoS One.

[44] Kim YS Issue paper series: labour and society: the non-regular work in South Korea. Bibliothek der Friedrich-Ebert-Stiftung.

[45] Pathirana TI, Jackson CA (2018). Socioeconomic status and multimorbidity: a systematic review and meta-analysis. Aust N Z J Public Health.

[46] Wang D, Li D, Mishra SR, Lim C, Dai X, Chen S, Xu X (2022). Association between marital relationship and multimorbidity in middle-aged adults: a longitudinal study across the US, UK, Europe, and China. Maturitas.

[47] Chudasama YV, Khunti KK, Zaccardi F, Rowlands AV, Yates T, Gillies CL, Davies MJ, Dhalwani NN (2019). Physical activity, multimorbidity, and life expectancy: a UK Biobank longitudinal study. BMC Med.

[48] Ng SK, Tawiah R, Sawyer M, Scuffham P (2018). Patterns of multimorbid health conditions: a systematic review of analytical methods and comparison analysis. Int J Epidemiol.

[49] Cheng W, Liu C, Hu K, Cheng Y, Karhula K, Härmä Mikko (2021). Night shift work and the risk of metabolic syndrome: findings from an 8-year hospital cohort. PLoS One.

[50] Vyas MV, Garg AX, Iansavichus AV, Costella J, Donner A, Laugsand LE, Janszky I, Mrkobrada M, Parraga G, Hackam DG (2012). Shift work and vascular events: systematic review and meta-analysis. BMJ.

[51] Ding C, Cicuttini F, Boon C, Boon P, Srikanth V, Cooley H, Jones G (2010). Knee and hip radiographic osteoarthritis predict total hip bone loss in older adults: a prospective study. J Bone Miner Res.

[52] Pouresmaeili F, Kamalidehghan B, Kamarehei M, Goh YM (2018). A comprehensive overview on osteoporosis and its risk factors. Ther Clin Risk Manag.

[53] Zhang Y, Jordan JM (2010). Epidemiology of osteoarthritis. Clin Geriatr Med.

[54] Boyd CM, Kent DM (2014). Evidence-based medicine and the hard problem of multimorbidity. J Gen Intern Med.

[55] Korea National Health and Nutrition Examination Survey.

