# Supplementary material for: Multimorbidity and its Associated Factors in Korean Shift Workers: Population-Based Cross-Sectional Study
Source: JMIR Public Health Surveill. 2024 Jun 10;10:e55014. doi: 10.2196/55014 (PMC11196912; doi:10.2196/55014)
Supplement: Multimedia Appendix 1 [file publichealth_v10i1e55014_app1.docx]

2016–2020 KNHANES participants

∙ 2016: *n* = 8,150 ∙ 2017: *n* = 8,127

∙ 2018: *n* = 7,992 ∙ 2019: *n* = 8,110

∙ 2020: *n* = 7,359

(Total *N* = 39,783)

Excluded (*n* = 7,610) due to age younger than 19

Adults aged 19 and older

(*n* = 32,128)

Excluded (*n* = 14,628) due to economically inactive or non-workers

Adult workers

(*n* = 17,500)

Excluded (*n* = 14,695) due to day workers

Adult shift workers

(*n* = 2,805)

Excluded (*n* = 1,101) due to missing patient data on chronic diseases

Final sample

(*n* = 1,704)

Appendix 1 Figure. The flow diagram of the study sample selection.
