# Supplementary material for: Multimorbidity and its Associated Factors in Korean Shift Workers: Population-Based Cross-Sectional Study
Source: JMIR Public Health Surveill. 2024 Jun 10;10:e55014. doi: 10.2196/55014 (PMC11196912; doi:10.2196/55014)
Supplement: Multimedia Appendix 2 [file publichealth_v10i1e55014_app2.docx]

Appendix 2 Table. Age-Subgroup Analysis of Factors affecting Multimorbidity in Shift Workers

(*N* = 1,704, *Weighted N* = 2,697,228)

| Subgroup | Younger than 50 years old age | | | 50 years or older age | | |
| --- | --- | --- | --- | --- | --- | --- |
|  | n=1,001 | | | n=703 | | |
| Factors | AOR | 95% CI | *P* value | AOR | 95% CI | *P* value |
| **Age** | **1.193** | **1.093 to 1.302** | **<.001** | 1.032 | 0.995 to 1.072 | .09 |
| House income (Quartile) |  |  |  |  |  |  |
| Low (1Q) | Ref. |  |  |  |  |  |
| Lower middle (2Q) | 0.606 | 0.113 to 3.240 | .56 | 0.548 | 0.290 to 1.037 | .06 |
| Higher middle (3Q) | 0.321 | 0.058 to 1.772 | .19 | **0.484** | **0.238 to 0.982** | **.045** |
| High (4Q) | 0.497 | 0.082 to 3.010 | .45 | 0.622 | 0.301 to 1.284 | .20 |
| Education |  |  |  |  |  |  |
| ≤ Elementary school | Ref. |  |  |  |  |  |
| Middle school | 1.015 | 0.090 to 11.415 | .99 | 0.532 | 0.275 to 1.026 | .06 |
| High school | 1.287 | 0.566 to 2.930 | .55 | **0.470** | **0.250 to 0.884** | **.02** |
| ≥ University | - | - | - | **0.303** | **0.147 to 0.626** | **.001** |
| Marital status |  |  |  |  |  |  |
| Married | Ref. |  |  |  |  |  |
| Single | 0.845 | 0.202 to 3.537 | .82 | **5.462** | **1.380 to 21.621** | **.016** |
| Regular work |  |  |  |  |  |  |
| Yes | Ref. |  |  |  |  |  |
| **No** | **4.233** | **1.188 to 15.075** | **.03** | 1.326 | 0.759 to 2.318 | .32 |
| BMI |  |  |  |  |  |  |
| Normal | Ref. |  |  |  |  |  |
| Underweight | - | - | - | 0.513 | 0.107 to 2.470 | .40 |
| Overweight | 0.494 | 0.094 to 2.601 | .40 | 1.081 | 0.586 to 1.995 | .80 |
| **Obese** | **3.174** | **1.100 to 9.157** | **.03** | **2.267** | **1.260 to 4.077** | **.007** |
| Smoking |  |  |  |  |  |  |
| Non-smoker | Ref. |  |  |  |  |  |
| Ex-smoker | 2.877 | 0.831 to 9.958 | .01 | 0.785 | 0.450 to 1.370 | .39 |
| Current smoker | 2.004 | 0.678 to 5.924 | .21 | 0.742 | 0.362 to 1.520 | .41 |
| Physical activity |  |  |  |  |  |  |
| No | Ref. |  |  |  |  |  |
| Yes | 1.237 | 0.503 to 3.040 | .64 | 1.025 | 0.630 to 1.666 | .92 |
| Poor sleep |  |  |  |  |  |  |
| No (7 to 9 h) | Ref. |  |  |  |  |  |
| Yes (< 7 h or > 9 h) | 2.177 | 0.832 to 5.695 | .11 | 1.221 | 0.760 to 1.961 | .41 |

Notes. AOR = adjusted odds ratio, CI = confidence interval, Ref = reference.

Bold face means statistically significant at p < 0.05.
