# Supplementary material for: Multimorbidity and its Associated Factors in Korean Shift Workers: Population-Based Cross-Sectional Study
Source: JMIR Public Health Surveill. 2024 Jun 10;10:e55014. doi: 10.2196/55014 (PMC11196912; doi:10.2196/55014)
Supplement: Multimedia Appendix 3 [file publichealth_v10i1e55014_app3.docx]

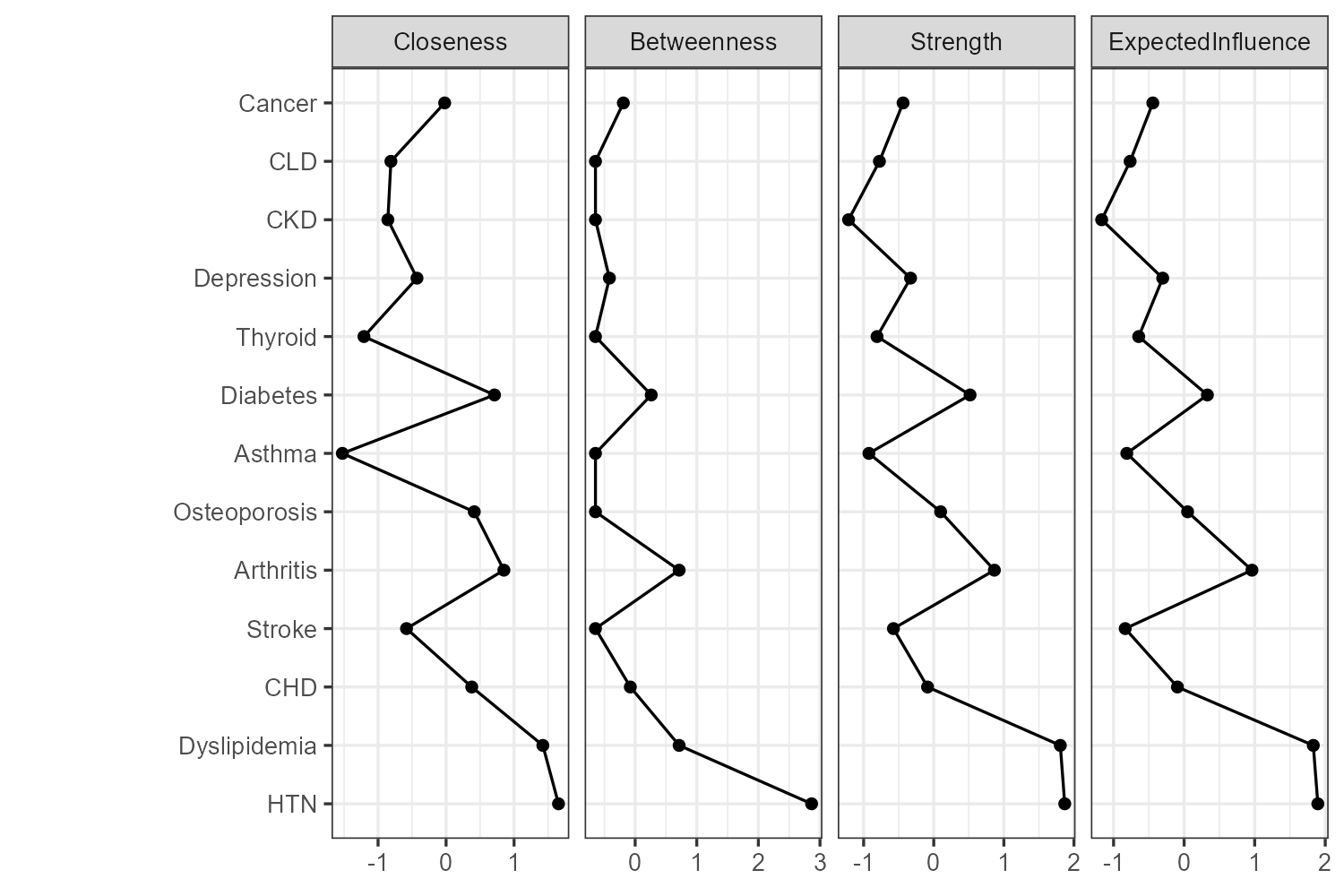


Closeness

Betweenness

Strength

Expected Influence

Appendix 3 Figure. Centrality plot of multimorbidity patterns in shift workers. Centrality plot of multimorbidity patterns in shift workers presents HTN, dyslipidemia, arthritis, diabetes, and osteoporosis according to strength and expected influence in order.
